# Supplementary material for: Open access publishers: The new players
Source: PLoS One. 2020 Jun 5;15(6):e0233432. doi: 10.1371/journal.pone.0233432 (PMC7274412; doi:10.1371/journal.pone.0233432)
Supplement: Attachment — Submitted filename: Response-to-reviewers.docx [file pone.0233432.s001.docx]

**PONE-D-20-00381 - Open access publishers: the DOAJ Seal profile

Response to reviewers**

1. Please consider changing the title so as to meet our title format requirement (https://journals.plos.org/plosone/s/submission-guidelines). In particular, the title should be "Specific, descriptive, concise, and comprehensible to readers outside the field" and in this case it is not informative and specific about your study's scope and methodology.

**We have changed it. We propose three options but we are open to new suggestions coming from the reviewers or the editors.**

2. We note you have included tables to which you do not refer in the text of your manuscript. Please ensure that you refer to Tables 2,3,5,6 in your text; if accepted, production will need this reference to link the reader to the Table.

**Revised.**

Reviewer #1

There is some interesting analysis here that adds to the understanding of open access and DOAJ’s role in helping identify high quality OA journals. The paper seems incomplete, however. Additional analysis, substantiation of several claims, and expanding the description of publishers would make this a better paper.

**Ok, we answer your comments below.**

In addition, the introduction to DOAJ should be expanded for those readers who are not familiar with it. See some details below.

**We have added more details about DOAJ (line 113).**

Lines 176, 177, 178. This statement is confusing and unsubstantiated. I suggest deleting it. You can just end the paragraph without it or find and cite a source that offers a substantiated version of what you are trying to say about novelty. Equating lack of novelty with higher quality and duplication confuses issues as it is stated. If the statement is rewritten and a source cited that supports it, note that “brings” should be “bring” in line 177.

**Deleted**.

In the introduction you mention that 3 large commercial publishers dominate the publishing industry/number of titles. In the findings you mention that PLOS and Biomed Central, MDPI and Hindawi in addition to Springer dominate the findings. You need to describe these publishers more and earlier in terms of commercial or non-profit, subject matter, etc. I would suggest expanding the introductory section on publishers to give more details rather than just those top 3 so the publishers you mention in the findings are not completely new to the reader when we reach this point. Perhaps a table of publishers would help. (For example, an extension of Table 1 in <https://www.stm-assoc.org/2018_10_04_STM_Report_2018.pdf>

**We have included a list of the current biggest academic publishers according to Ulrich’s and a new paragraph analyzing the most prominent open access publishers (line 71).**

Lines 200+ you mention some characteristics, but information about publishers is scattered, so this introduction would help readers put these scattered statements into context.

**We agree on that, thanks.**

Your Table 1: should be titled “…by the Biggest Open Access Publishers”

**Added**.

It seems that Table 2 should logically come before Table 1.

**We do not agree with that. We think that table 1 is more general and offers a global overview of the publisher’s scenario. Table 2 includes just the years.**

Lines 257-260: This comment is unsubstantiated and should be supported or deleted. It is jarring and seemingly irrelevant. 257 “This highlights what appears 258 to be the real motivation behind the creation of new journals in Open Access: the 259 company’s portfolio interests in the market, rather than the needs of society or the 260 pursuit of scientific innovation.”

**Deleted.**

Line 269: Also see: Shen, C., & Björk, B.-C. (2015). ‘Predatory’ open access: a longitudinal study
of article volumes and market characteristics. BMC Medicine, 13(230), 1-15. doi:
10.1186/s12916-015-0469-2
You should expand the discussion of predatory OA and how DOAJ hopes to provide a “white list” that eliminates those.

**We have added this reference beneath a previous one (Somoza et al, 2018). We have included also a comment about the role of DOAJ against predatory journals (“white list”).**

Since in your conclusion (lines 283-285) you make a bold prediction (283: “The high concentration of journals controlled by commercial publishers may generate a price crisis in the Open Access movement similar to that of the 1960s, when prices rose so high that libraries were forced to cancel subscriptions.”) you need to include analysis on prices. This statement again needs evidence and your study is in a position to do so, or at least include references and a discussion of price studies by others.

**We have changed the sentence and the argument and we have added the reference to Khoo, S.Y.-S., 2019.**

In summary: include characteristics of publishers in introductory section, remove or substantiate several statements that are not substantiated by evidence from data analysis or literature review, add to the literature and discussion on predatory journals and how DOAJ serves as a white list to help authors select reputable OA journals.

**Ok, thanks for your comments.**

Reviewer #2:

This manuscript represents an interesting analysis of the publishers business model and journals' behaviour respect to some critical variables in the OA era. Focusing on the DOAJ seal is a good point of analysis. The overall manuscript is well-written, the aims of the study and the conclusions are consistent. Just a few observations:

2.Methodology section: when you give numbers, please specify the date of your search as nowaday the numbers are different, of course, even if slightly.

**Changed (line 127 … “in March of 2019”.)**

3.1: the observation of lines 210-212 deserves to be stressed also in the conclusion section because is very meaningful!

**We have added a comment in the conclusions (line 440)**

3.2 Distribution of knowledge areas, line 257-260: this statement is more a consideration than a description. you could put it in the Results and Discussion section.

**We agree on that, but this statement is now in the Results and Discussion section. This consideration or reflection is based on the data collected and starts a relevant discussion.**

Just a final observation: discussing about PLOSone in PLOSone...I think your analysis is quite objective but... in conclusion, it's a manuscript, very interesting to be read!

**Thanks for your comments. We have tried to discuss about open access publishers and PLOSOne is part of this reality. We think also that it is a good journal to send our manuscript and for improving it (correcting any bias or problem) with the help of our reviewers.**
