# Supplementary material for: Open access publishers: The new players
Source: PLoS One. 2020 Jun 5;15(6):e0233432. doi: 10.1371/journal.pone.0233432 (PMC7274412; doi:10.1371/journal.pone.0233432)
Supplement: Attachment — Submitted filename: Response-to-reviewers.docx [file pone.0233432.s002.docx]

**PONE-D-20-00381R1: Open access publishers: the new players**

**Response to reviewers**

**Reviewer #1: Thank you for addressing all of the comments. By making these changes the paper is now stronger. This is an interesting paper.**
Many thanks for your remarkable comments which have contribute to improve the paper prominently.

**Reviewer #2: Interesting paper,**

Many thanks for your remarkable comments which have contribute to improve the paper prominently.

**the observations well considered, just some misspelling at lines 89 - 4 Not 5 type of OA –**

Changed.

**148, 154**.
We’re sorry but we haven’t found the mistakes in these lines. We hope that the copyeditor will find them.

**The conclusions are consistent with the aims of the study but, at the end, the DOAJ seal seems forgotten... I would recommend to remind readers that we are in this context...**

Changed, we’ve included a remind.
